# The Brachypterous Species of *Passalus* (*Pertinax*) (Coleoptera: Passalidae), with the Description of a New Species from Sierra Nevada de Santa Marta, Colombia

**DOI:** 10.1007/s13744-022-00988-1

**Published:** 2022-09-21

**Authors:** Larry Jiménez-Ferbans, Pedro Reyes-Castillo, Marcus Bevilaqua

**Affiliations:** 1 Facultad de Ciencias Básicas, Universidad del Magdalena, Santa Marta, Colombia; 2 Red de Biodiversidad Y Sistemática, Instituto de Ecología, A.C., Xalapa-Enríquez, Mexico; 3 Lab de Sistemática E Ecologia de Coleoptera, Instituto Nacional de Pesquisas da Amazônia, Manaus, Brazil

**Keywords:** Bess beetles, Passalids, Taxonomy, Brachypterism

## Abstract

Brachypterism is a common condition cited in passalid beetles, mainly associated to species with strictly montane distributions. In the New World Passalids, brachypterism has been reported especially in the tribe Proculini, for which almost all genera have brachypterous species; meanwhile, in Passalini, the other Netropical tribe, it has been cited only for the genus *Passalus*, mainly in the subgenus *Passalus* (*Passalus*). Here we present a commented list of the brachypterous species of the subgenus *Passalus* (*Pertinax*); we redescribed *Passalus gravelyi* Moreira, *P*. *quitensis* (Kaup), *P*. *striatissimus* Luederwaldt, and *P. sulcifrons* (Kuwert) and describe a new species from Sierra Nevada de Santa Marta, Colombia.

## Introduction

Brachypterism (reduction of membranous wings) is a relatively common phenomenon within Coleoptera, and in Passalidae it is present in several independent lineages (Reyes-Castillo 1970; Boucher 2006). In other families, the wing reduction can reach complete atrophy (e.g., Carabidae); however, in Passalidae complete atrophy does not happen, maybe because the hind wing has a dual function: flight and stridulation. Due to its subsocial behavior, in Passalidae the function of flight is of less importance than that of stridulation, the latter being favored by the wing reduction (Reyes-Castillo 1970).

**Fig. 1:**
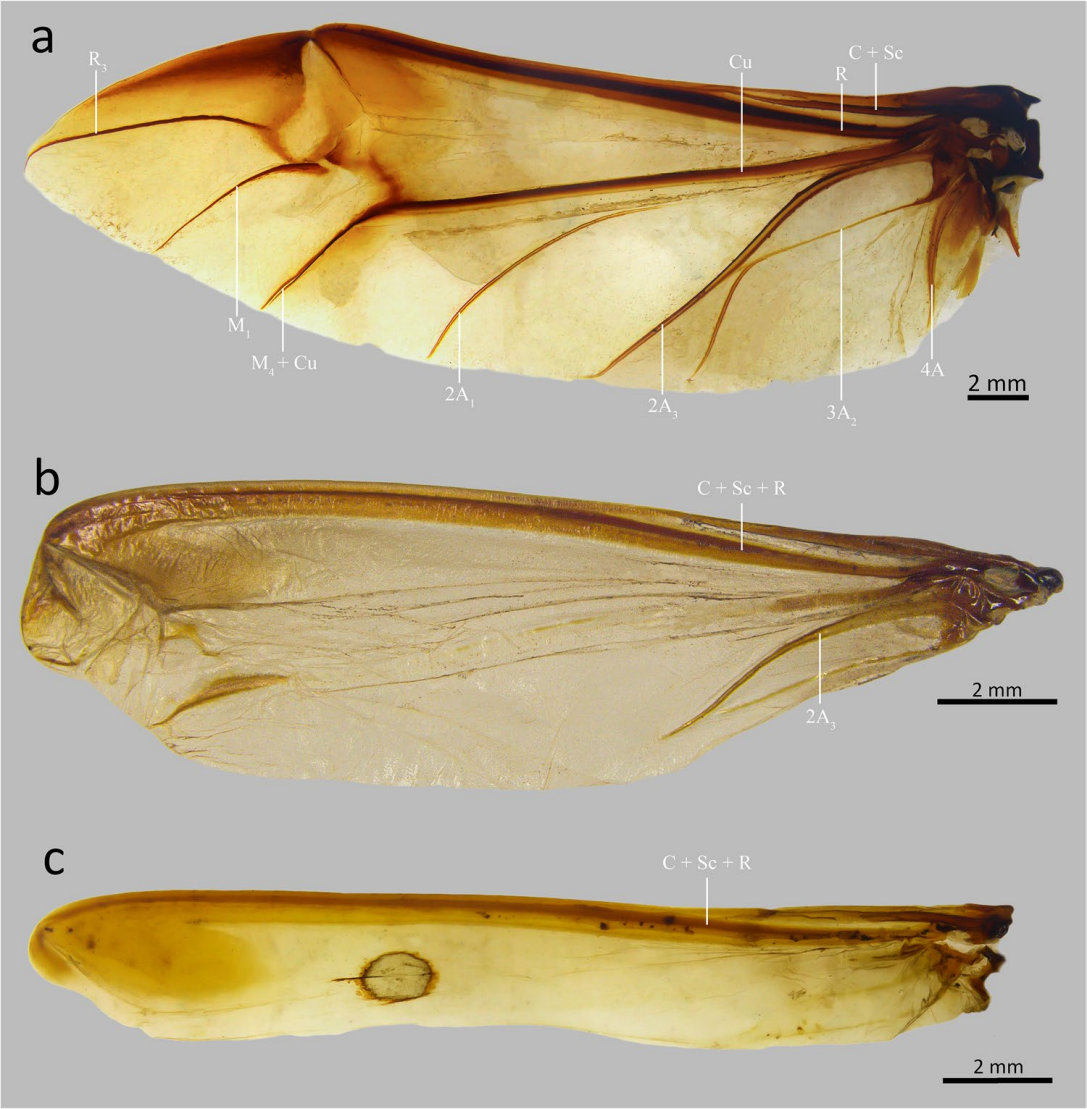
Wings of (**a**) *Passalus convexus* Dalman, macropterous; (**b**) *Passalus gaboi*, hemibrachypterous; (**c**) *Passalus gonzalezae*, brachypterous

In Passalidae the degree of wing reduction is variable, a completely reduced wing is shorter than the length of the elytra, narrow at the proximal region and gradually expanded to the distal region. There is a complete absence of anal veins, M_1_ and R_3_; the fusion of the costal, subcostal, and radial veins is remarkable; the flexion point of the wing almost disappears and moves to the middle third of the distal edge. The reduced wing has a more coriaceous surface and undergoes a strong sclerotization of the apical part, which is the stridulation zone (Reyes-Castillo 1970). According to Boucher (2006), the distinction between a normal and reduced wing can be made by the ratio between the length of the unfolded wing and the total length of the body. A proportion of 1/1 to 4/5 corresponds to a specimen with normal wings and 2/3 to 1/2 corresponds to a specimen with reduced wings. The term hemibrachypterous has been used to indicate “intermediate” alar reduction, between brachypterism and macropterism (Boucher 2006); however, its definition is ambiguous. To us, a species can be considered hemibrachypterous when the radial and anal veins of the wing are still preserved, but reduced (Fig. 1).

Although most known cases of brachyptery occur in Neotropical species of Passalidae (mainly in the tribe Proculini), there are also Ethiopian, Oriental, and Australian brachypterous species (Hincks 1933), this characteristic being apparently related to the habit of living in mountains (Reyes-Castillo 1970; Ariza-Marín and Amat-García 2021). In Proculini, which originated in the mountains of Mesoamerican (Beza-Beza et al. 2021), this condition seems to be more widespread, with almost all genera (16 of 20) having brachypterous species and some genera comprised entirely of flightless species (e.g., *Ogyges*, *Proculejus*, and *Proculus*). Meanwhile, in Passalini, which is postulated to have originated in South America (Reyes-Castillo and Halffter 1978; Fonseca 1987), of the seven genera (Jiménez-Ferbans et al. 2022) only *Passalus* contains brachypterous species.

*Passalus* comprises 171 species: 81 in *P*. (*Pertinax*), 11 in *P.* (*Mitrorhinus*), and 79 in *P*. (*Passalus*) (Bevilaqua and Fonseca 2020). *Passalus* (*Mitrorhinus*) does not have flightless species, while in *P*. (*Passalus*) the brachypterous species are in the ‘Petrejus’ group; of the 33 species, about 6 have wing reduction, although the correct number may be higher, and in *Passalus* (*Pertinax*), 11 of its 81 species are brachypterous. Hitherto, no brachypterous species of *Passalus* (*Pertinax*) is known from Colombia, which is the country with more species of Passalidae (Jiménez-Ferbans et al. 2018). Here we present a commented list of the brachypterous species of this subgenus, redescribe *Passalus gravelyi*, *P*. *quitensis*, and *P*. *striatissimus*, and describe a new species from Sierra Nevada de Santa Marta, Colombia.

## Materials and methods

We reviewed the descriptions for species of *Passalus* (*Pertinax*) and studied specimens deposited in the Entomological Collection Universidad del Magdalena (CEBUMAG-ENT, Colombia); the Entomological Collection of the Institute of Ecology (IEXA, Mexico); the Entomological Collection of the Instituto Nacional de Pesquisas da Amazônia (INPA, Manaus, Brazil); the Museu de Zoologia, Universidade de São Paulo (MZUSP, São Paulo, Brazil); the Insect Collection, Federal University of Mato Grosso (CEMT, Cuiabá, Brazil); the Natural History Museum (BMNH, London, UK); the Manchester Museum, University of Manchester (MMUE, Manchester, UK); and the Oregon State Arthropod Collection (OSAC). The terminology employed for the head is the proposed terminology by Boucher (2006), for the rest of the body that of Reyes-Castillo (1970).

The images for *Passalus gaboi* n.sp. were obtained with a Leica M205A motorized. For the rest of the species, the images were obtained in a Leica M165C stereomicroscope with a coupled DFC295 camera and processed in the LAS version 4.2 or by a Zeiss AxioCam MRc 5 video camera attached to a Carl Zeiss Discovery stereomicroscope. Then, they were stacked in layers by the software Helicon Focus version 7.6.1 to generate a single image of combined focus. For uniform and more effective illumination, we used the geodesic dome of lighting according to Kawada & Buffington (2016). Illustrations were produced via camera lucida attached to stereomicroscopes. Illustrations and photographs were edited for light and contrast correction in Adobe Photoshop, and the figures were made using the same software, following the guide proposed by Bevilaqua (2020). The map for the distributional records was created with QGIS software version 3.18 (available at http://www.qgis.org). Localities in the map are approximate localities obtained from specimen labels and published literature sources.

## Results

### Taxonomy

**Family PASSALIDAE Leach, 1815.**

**Subfamily PASSALINAE Leach, 1815.**

**Genus** *Passalus* Fabricius, 1792.

**Subgenus** *Passalus (Pertinax)* Kaup, 1869.

## The brachypterous species of *Passalus* (*Pertinax*)

### *Passalus* (*Pertinax*) *gaboi* n.sp. Jiménez-Ferbans & Reyes-Castillo (Figs. 1, 2 and 3)

Type material. Holotype: ♂ **Colombia**. Magdalena. Santa Marta. Sierra Nevada de Santa Marta. San Lorenzo. 11°06′21.7″N 74°04′09.1″W. 2069 msnm. 13.v.2018. Col.: Jiménez-Ferbans et al. / Punto 12 Tronco 2 (CBUMAG-ENT).

**Fig. 2:**
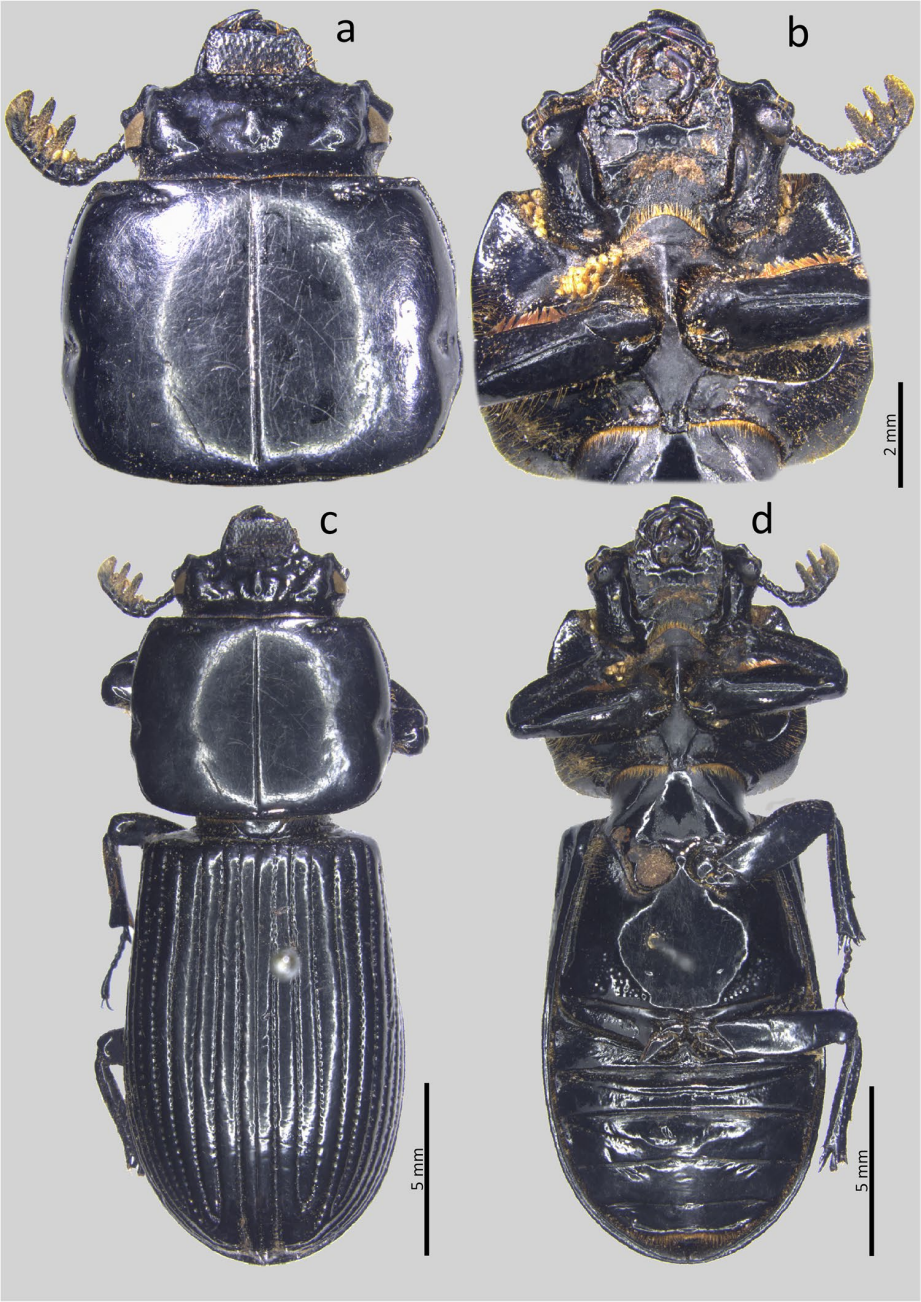
*Passalus* (*Pertinax*) *gaboi* n.sp.: (**a**) head and pronotum in dorsal view; (**b**) head and prosternum in ventral view; (**c**) habitus dorsal; (**d**) habitus ventral

**Fig. 3:**
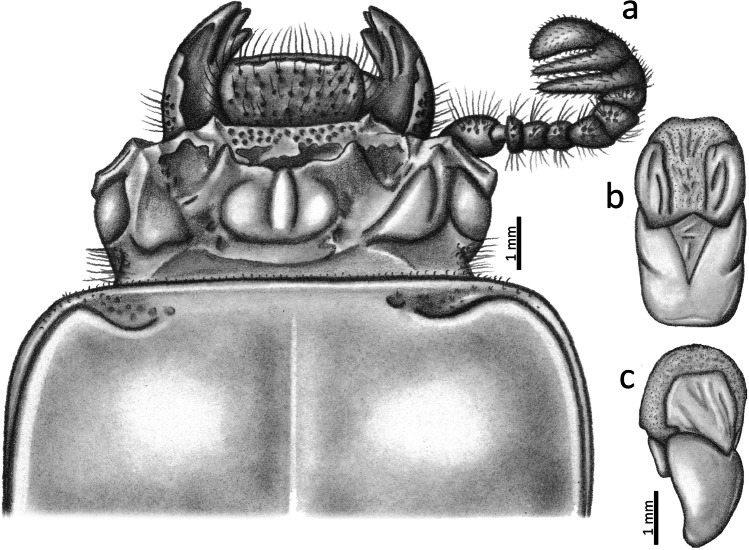
*Passalus* (*Pertinax*) *gaboi* n.sp.: (**a**) head and pronotum in dorsal view; (**b**) aedeagus in ventral view; (**c**) aedeagus in lateral view

Paratypes: **Colombia**: Magdalena. Santa Marta. Sierra Nevada de Santa Marta. 11.10222, -74.06974. 2059 msnm. 5.vi.2015. Col.: Pedro Reyes, Jose Pérez, Andres Rocha, Luis Rueda (1♀, 1♂, CBUMAG-ENT, IEXA). Magdalena. Santa Marta. Sierra Nevada de Santa Marta. San Lorenzo. 11°06′21.7″N 74°04′09.1″W. 2069 msnm. 13.v.2018. Col.: Jiménez-Ferbans et al. // *Passalus gaboi* sp.nov1. det. Jiménez-Ferbans (CBUMAG-ENT). Magdalena. Santa Marta. Sierra Nevada de Santa Marta. San Lorenzo. 11°06′21.7″N 74°04′09.1″W. 2069 msnm. 13.v.2018. Col.: Jiménez-Ferbans et al. / Punto 12 Tronco 2 (2♀♀, CBUMAG-ENT). Magdalena. Santa Marta. Sierra Nevada de Santa Marta. San Lorenzo. 11°06′11.3″N 74°04′18.4″W. 1960 msnm. 13.v.2018. Col.: Jiménez-Ferbans et al. (♂, CBUMAG-ENT). Magdalena. Santa Marta. S.N.S.M. San Lorenzo. 11°06′11.5″N 74°04′18.1″W. 1938 msnm. 13.v.2018. P7.T1. Col.: Jiménez-Ferbans. // *Passalus* (*Pertinax*) *gaboi* n.sp. Det.: Jiménez-Ferbans, 2018 // PARATYPE (1, OSAC). Magdalena. Santa Marta. SNSM. San Lorenzo. 11°06′21.6″N 74°04′08.6″W. 2040 msnm. 21.vi.2019. Col.: Jiménez-Ferbans // *Passalus gaboi* det. Jiménez-Ferbans (1♂, CBUMAG-ENT). Magdalena. Santa Marta. SNSM. San Lorenzo. 11°06′29.6″N 74°04′15.3″W. 2095 msnm. 21.vi.2019. Caminando a la orilla de la carretera. Col.: Jiménez-Ferbans // *Passalus gaboi* det. Jiménez-Ferbans (1♂, CBUMAG-ENT). Magdalena. Santa Marta. SNSM. San Lorenzo. 11°06.608′N 74°03.658′W. 2190 msnm. 19.vi.2019. Col.: Jiménez-Ferbans // *Passalus gaboi* det. Jiménez-Ferbans (CBUMAG-ENT).

Zoobank registration number: 6109A2CC-D1B4-4112–9513-7F1D4DBF4770.

Diagnosis: hemibrachypterous; anterior frontal edge with small middle indentation; area mediofrontal heavily punctuated on the anterior half, divided by a longitudinal sulcus which reaches to the base of the central tubercle, the last one with apex not free. mediofrontal + laterofrontal tubercles same size than internal tubercles.

Description.

Habitus (Figs. 2c-d) : total length 32.8–34.2 mm, hemibrachypterous, body convex shiny black.

Head (Figs. 2a-b and 3a): labrum with anterior border almost straight, with a small medial projection, evenly covered by setae. Clypeus hidden under the frons, anterior angles developed under the mediofrontal + laterofrontal tubercles and slightly smaller than these. Frons wide, anterior frontal edge with small middle indentation, without secondary mediofrontal tubercles. Mediofrontal + laterofrontal tubercles projected forward, midsize, same size than internal tubercles. Internal tubercles big, projected upwards, not joined to mediofrontal + laterofrontal tubercles by ridge, placed at mid distance between the mediofrontal tubercles and the central tubercle apex. Posterofrontal ridges “V” shaped. Area between the frontal ridges heavily punctuated on the anterior half, divided by a sulcus that extends from the anterior border to the base of the central tubercle. Cephalic mamelon (sensu Jiménez-Ferbans & Reyes-Castillo 2014) present and divided. Mesofrontal structure of the “marginatus” type (Reyes-Castillo 1970), with central tubercle wide at the base, without a sulcus in the posterior part, apex not free. Lateroposterior tubercles slightly marked and rounded. Lateropostfrontal areas glabrous, shiny, and punctate posteriorly. Eyes reduced, with canthus covering ½ of the eye in lateral view. Canthus glabrous. Postorbital pits weak. Postfrontal groove semicircular and complete. Hypostomal process slightly separated from mentum, glabrous and reaching the superior part of the middle zone of the mentum. Medial basal mentum protruding ventrally, heavily punctate and pubescent. Mentum with big lateral fossae, shallow and pubescent. Antennal club trilamellate. Dorsal tooth straight on dorsal view and slightly sinuous on lateral view. Dorsal mandibular pubescence covering the base of the mobile tooth, not reaching the base of the internal tooth. Internal tooth of the left mandible bidentate, simple on the right mandible. Mandibular fossae short, not reaching the base of the mobile tooth. Maxilla with lacinia bidentate at the apex. Ligula tridentate, with middle tooth longer than lateral teeth. Middle palpomere of the labial palp 1.3 times wider and with almost the same length as the distal palpomere.

Thorax (Figs. 2a-b): Pronotum rounded, wider than elytra, with punctuations restricted to the lateral fossae and marginal groove. Marginal groove wide, occupying ¾ of the anterior margin of the pronotum. Longitudinal sulcus well marked. Lateral fossae marked. Prepimerum opaque and heavily pubescent. Prosternellum rhomboidal and opaque. Mesosternum without mesosternal scars, indicated only by an opaque area, impunctate and glabrous. Posterior corner of the mesepisternum and mesepimere glabrous. Anterolateral part of metasternum and lateral fossa pubescent. Metasternal disc without punctures, delimited by numerous punctures medially and posteriorly. Posterior metasternal lateral fossa of the same width as epipleura.

Elytra (Fig. 2c): Shiny, anterior border straight and glabrous. Humeri and Epipleura glabrous. Striae with rounded punctures, more marked on lateral striae.

Abdomen (Fig. 2d): Last sternite with marginal groove complete.

Legs (Figs. 2c-d): profemur with ventral anterior marginal sulcus thin and complete, reaching the apical pubescence. Protibiae with dorsal sulcus complete. Mesotibiae with small spines on the outer margin. Metatibiae unarmed.

Aedeagus (Figs. 3b-c): Basal piece (ventral view) fully fused with parameres and with deep “v”-shape cleft. Median lobe globose, little sclerotized on ventral surface, length is 1.1 times the length of the basal piece and parameres, measured at the median ventral line. Lateral projections of the parameres short and apex truncated on lateral view.

Variation: punctures on the area between the frontal ridges are surrounding the cephalic mamelón in the holotype, whereas they are only in the anterior part in the paratype. On the other hand, the middle indentation in the frons border is more marked in the paratypes than in the holotype.

### ***Passalus*** (***Pertinax***) ***gravelyi*** Moreira, 1922 (Figs. 4, 5 and 6)

Type material. Holotype: male. **Brazil**. [Rio de Janeiro—Itatiaia] // Instituto Biol. nº 43 // Inst. Biolog. Entomologia Agricola Rio de Janeiro // Laboratório de Entomologia Agricola Rio de Janeiro (MZUSP).

**Fig. 4:**
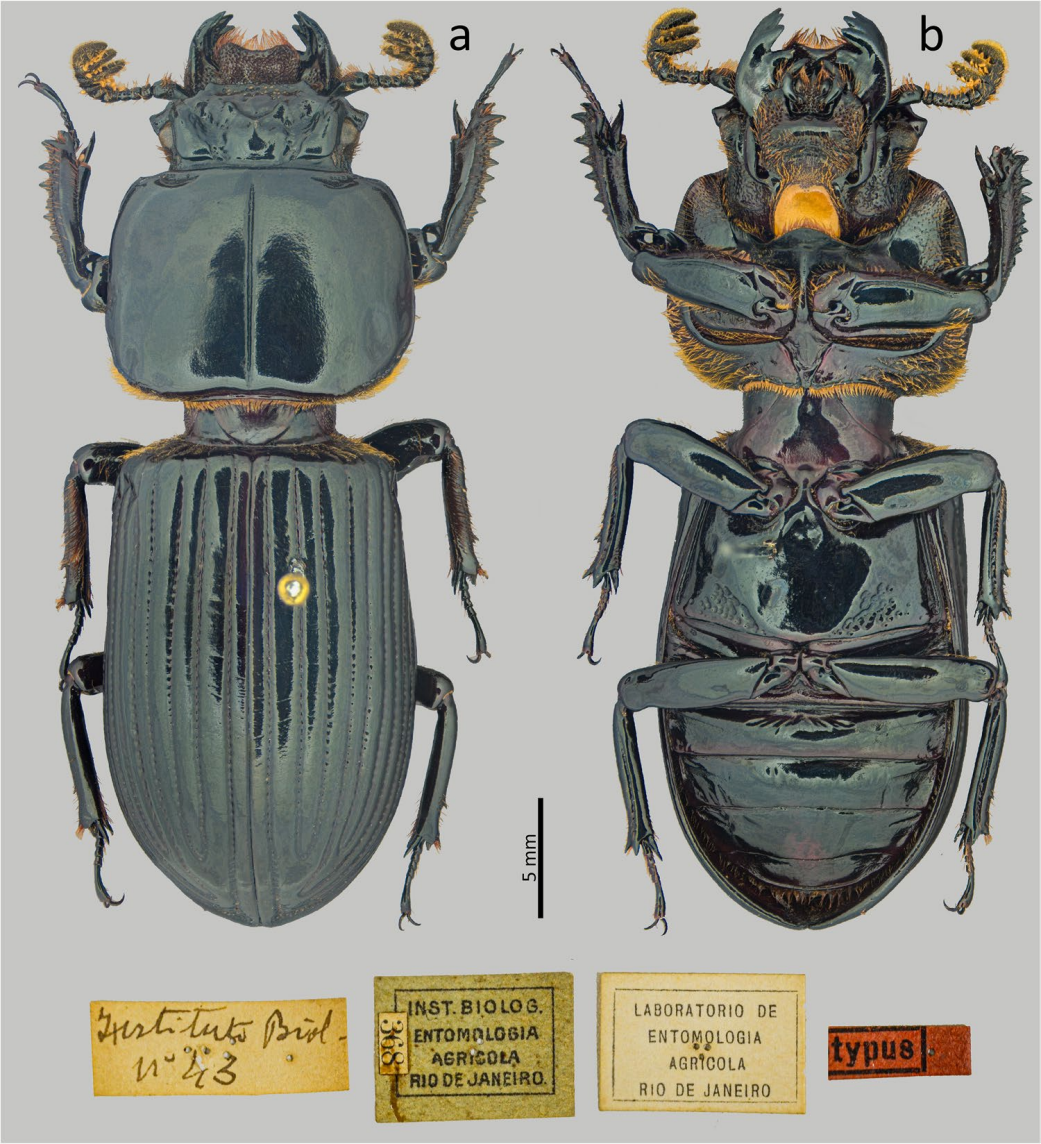
*Passalus* (*Pertinax*) *gravelyi* type: (**a**) dorsal habitus; (**b**) ventral habitus

**Fig. 5:**
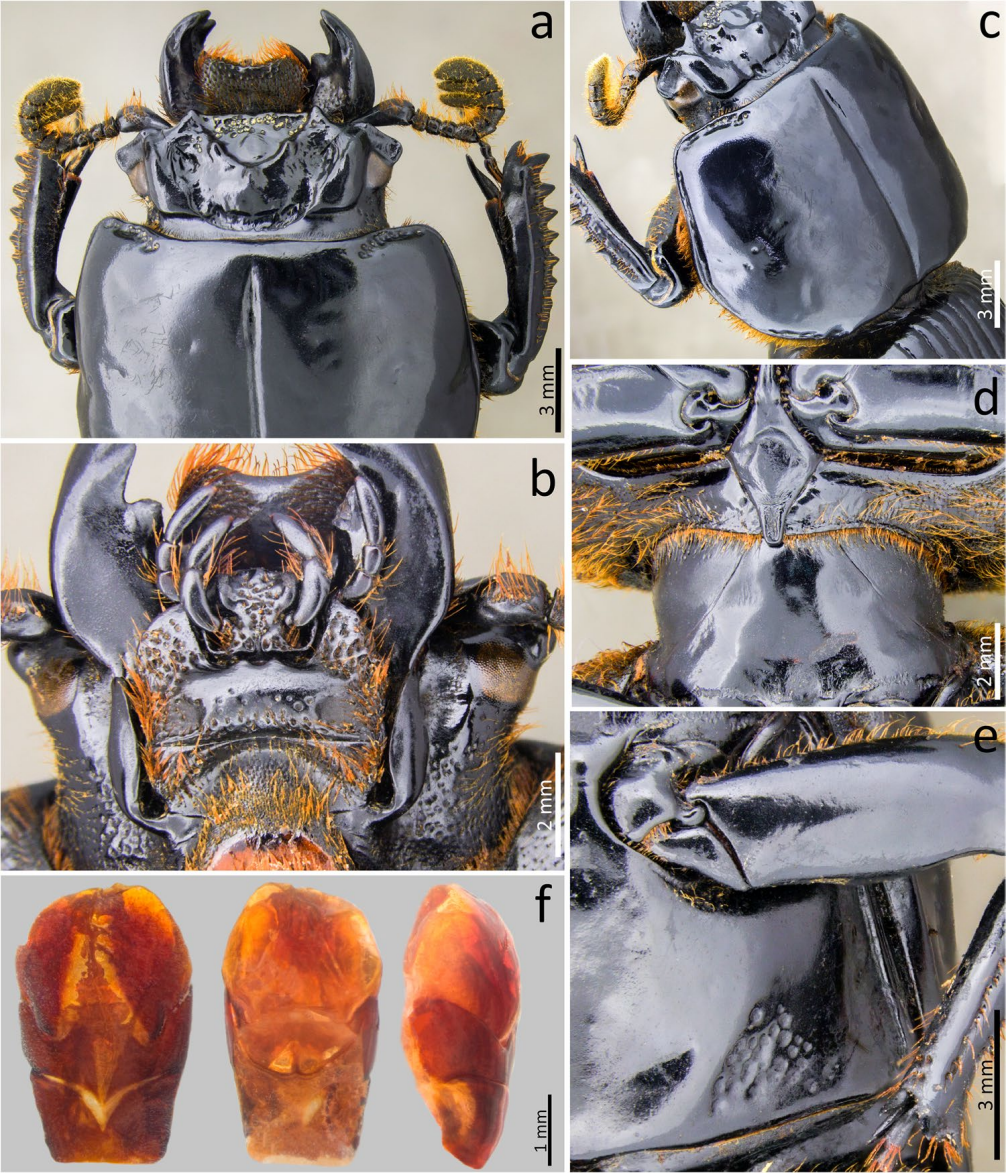
*Passalus* (*Pertinax*) *gravelyi:* (**a**) head; (**b**) mentum; (**c**) head and pronotum in dorsolateral view; (**d**) mesosternum; (**e**) metasternum; (**f**) aedeagus

**Fig. 6:**
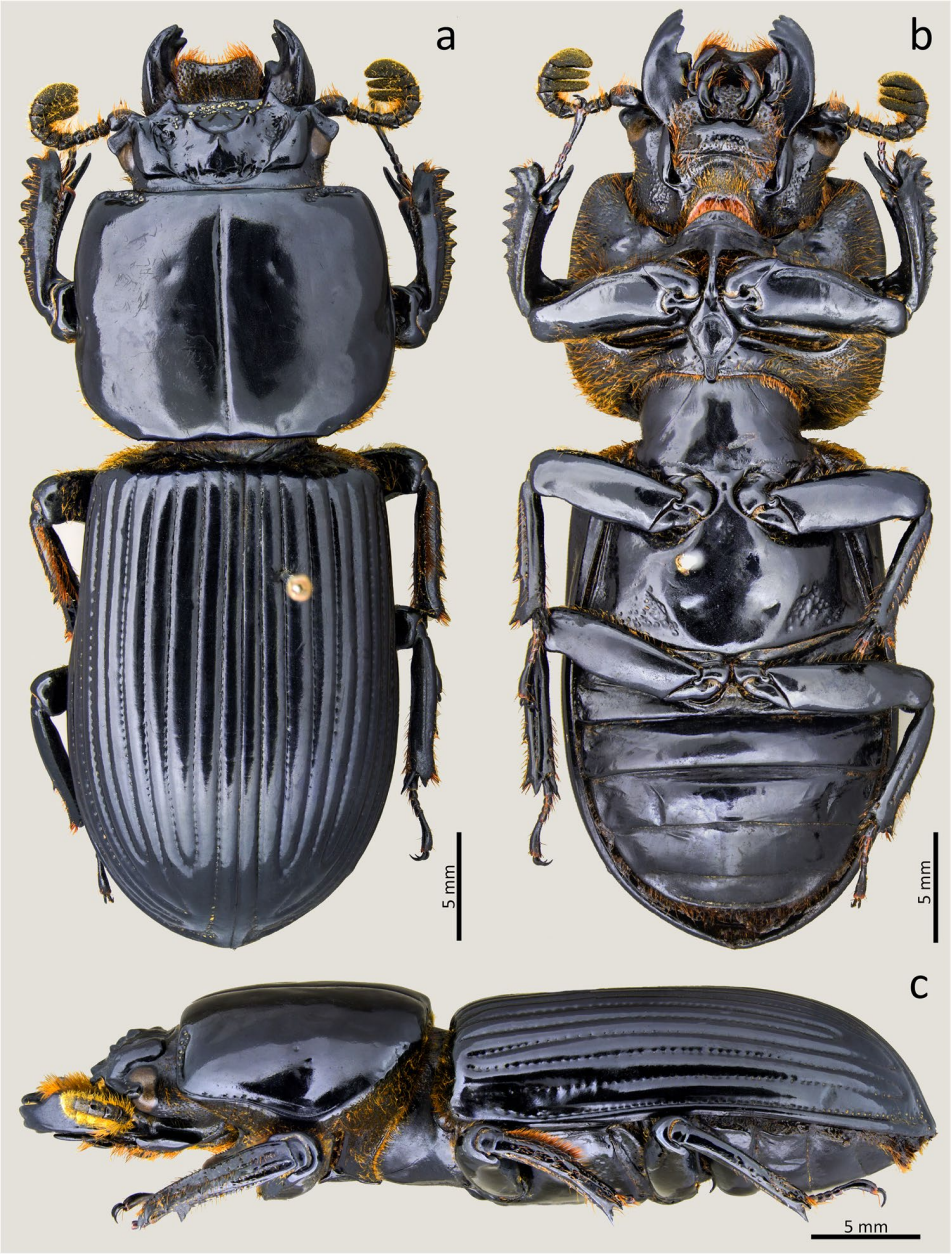
*Passalus* (*Pertinax*) *gravelyi*: (**a**) dorsal habitus; (**b**) ventral habitus; (**c**) lateral habitus

Additional material: **Brazil**. *Rio de Janeiro*: Itatiaia, Maromba, 1957 / F. H. Guimarães // *Passalus gravelyi* / P. Pereira det. [1]957. (MZUSP) 1 ex. Itatiaya 1800 m. xii.[19]30. W. Zikan leg. // *Passalus gravelyi* Mor. Lued. det. 1932 // 06,505 (MZUSP) 1 ex. Itatiaya, 1300 m, ii.1936 / B. Pohl // *Passalus* (*Pertinax*) *gravelyi* Moreira (MZUSP) 1 ex. [unleg.] 1920 Hoffmann // *Passalus gravelyi* Mor. Luederw. det. [19]28 // 06,506 (MZUSP) 1 ex. Itatiaya—1800 m. Km 13. 16.iii.1935 / J. F. Zikán // *P*. (*Pertinax*) *gravelyi* Moreira / J. F. Zikán det. // 05,589 (MZUSP) 1 ex. *Minas Gerais*: Sapucaí-Mirim. Cidade Azul—1400 m. 7.xi.1953 / L. Trav. F. & M. Kuhlmann, C. Gans & S. Medeiros // *Passalus gravelyi* / P. Pereira det. [1]954 // 20,428, 20,425, 20,427 (MZUSP) 3 ex. Vila Monte Verde 28.ii.1964. J. Halik / 897 // *Passalus gravelyi* Moreira / P. Pereira det. [1]964 (MZUSP) 1 ex. Vila Monte Verde 15.iii.1966 / J. Halik // *Passalus gravelyi* Mor. / P. Pereira det. [1]966. (MZUSP) 2 ex. Vila Monte Verde, 21.xi.1966 / J. Halik. 5052 // *Passalus* (*Pertinax*) *gravelyi* Moreira / Reyes-Castillo det. [19]88. (MZUSP) 1 ex. Vila Monte Verde, 10.xii.1966 / J. Halik 5051 // *Passalus gravelyi* / P. Pereira det. [1]967 (MZUSP) 1 ex. Vila Monte Verde, 12.viii.1968 / Halik 7624 // *Passalus gravelyi* Mor. / P. Pereira [1]968 (MZUSP) 1 ex. Virgínia. S[ul] Minas Ger[ais] Faz.[enda] Campos—1500 m. 24.ii.1915. J. F. Zikan // *Passalus gravelyi* Mor. Luederw. det. [19]28 // 06,503 (MZUSP) 1 ex. Virginia S.[ul] Minas Ger.[ais] Faz.[enda] Campos—1500 m. 23.xii.1917 / J. F. Zikán // *Passalus gravelyi* Mor. / Luederw. det. [19]28 // 06,504. (MZUSP) 1 ex. D.[elfim] Moreira [unleg.] de Piquête 1300 m. x.1957 // *Passalus gravelyi* (MZUSP) 1 ex. *São Paulo*: Campos do Jordão, 1600 m. ii.1958 / K. Lenko. (MZUSP) 1 ex. *Paraná*: Faz.[enda] Monte Alegre 858 m—vii.[1]941 / R. Lange leg. (MZUSP) 1 ex.

Diagnosis: Frons anterior edge straight, with small middle indentation; mediofrontal + laterofrontal tubercles large, with slightly obtuse apex; inner tubercles large, with acute vertices, smaller than the mediofrontal + laterofrontal tubercles; canthus ocular wide, eyes reduced; prepisternum thin, pubescent with long setae located mainly in the lateroposterior region; mesosternal scars absent or inconspicuous; humeri with short tuft of setae.

Redescription.

Habitus (Figs. 4 and 6): total length 39–42 mm, brachypterous, body convex.

Head (Figs. 5a-b): labrum with anterior border concave. Clypeus hidden below the frons, anterior angles small, positioned below the mediofrontal + laterofrontal tubercles. Frons anterior edge straight, with small middle indentation, without secondary mediofrontal tubercles. Mediofrontal + laterofrontal tubercles large, conspicuous, projected forward with slightly obtuse apex. Frontal area transverse, wider than long, sloping, with the tegument of the anterior region full of thick punctuations. Inner tubercles large, conspicuous, with acute vertices, smaller than the mediofrontal + laterofrontal tubercles, from which they are separated, located closer to central tubercle. Anterior frontal ridges absent. Posterior frontal ridges strong, low, starting at an obtuse angle at the base of the central tubercle. Cephalic mamelon (sensu Jiménez-Ferbans & Reyes-Castillo 2014) large, conspicuous, and divided by a weak longitudinal groove. Central tubercle conical, flattened, low posteriorly, with only the apex evident, but not free. Lateroposterior tubercles small, inconspicuous. Laterofrontal areas prominent and punctate. Lateropostfrontal areas glabrous and not punctate. Postfrontal groove well-marked, with notch in the middle region. Epicranial sutures well-marked. Epicranial fossae shallow but evident. Anterior head angles well-developed, with obtuse vertices, smaller than mediofrontal + laterofrontal tubercles. Canthus ocular wide, with straight anterior angles or slightly obtuse, reaching halfway to the eye (eyes reduced). Postorbital fossae slightly marked, deep, unpunctuated and with some setae. Hypostomal process broad, glabrous and slightly distant from mentum. Medial basal mentum slightly dilated, with few setigerous punctuations only in the posterior region; with protruded anterior region and notched. Lateral lobes of the mentum rounded outer face and straight inner face, with sparse setae. Mentum with big lateral fossae, shallow and some setae on the outer surface. Antennal club trilamellate, with short, thick, and straight lamellae. Mandibles with incisor lobe with three well-formed teeth at the apex; strong superior inner tooth; dorsal teeth large and tall, directed forward; inconspicuous infrabasal fossae. Maxilla with lacinia bidentate at the apex. Ligula tridentate with medium tooth slightly larger and with the same width as the lateral teeth. Thorax (Figs. 4, 5c-e and 6): pronotum rounded, without punctuations, same wide than elytra. Anterior edge slightly straight. Anterior angles obtuse. Marginal groove narrow, occupying ½ of the anterior margin of the pronotum, containing fine punctuations in the anterior part, practically without punctuations laterally. Lateral fossae large, well-marked, deep, irregular in shape. Prepisternum thin, pubescent with long setae located mainly in the lateroposterior region. Prepimerum densely pubescent with long setae, mainly in the posterior region, which can be visible in dorsal view. Prosternellum rhomboidal, with acute base. Mesosternum smooth and glabrous, with lateral areas tegument opaque. Mesosternal scars absent or inconspicuous. Posterior corner of the mesepisternum and mesepimere glabrous. Anterolateral part of metasternum and lateral fossa glabrous. Metasternal disc short and flat, not punctuated. Metasternal punctuations consisting in a group of thick punctures in the posterior region, not forming a carina that delimits the metasternal disc. Metasternal lateral fossea narrow, not dilated posteriorly, narrower than the mesotibia.

Elytra (Figs. 4a and 6): epipleura glabrous. Humeri with short tuft of setae. Striae narrower than interstriae; marked with shallow and inconspicuous punctuations on the dorsal striae and slightly more defined and deeper on the lateral striae.

Abdomen (Figs. 4b and 6b): Last sternite with marginal groove complete and well-marked.

Legs (Figs. 4 and 6): profemur with ventral anterior marginal sulcus marked and complete; posterior ventral edge with tuft of setae close to the apex. Protibiae not dilated. Mesotibiae with two or three small spines on the outer margin. Metatibiae without or with a small spine on the outer surface.

Aedeagus elongated (Fig. 5f). Basal piece slightly longer than the parameres, distinctly separated from the parameres, with “v”-shape cleft. Median lobe wider than the basal piece and parameres; in ventral view with two sclerotized plaques, with narrower posterior regions, almost acute. Parameres in ventral view distinctly medially separated, deeply emarginated in the anterior edge; in lateral view, with slightly straight projections not reaching half the length of the median lobe.

### ***Passalus*** (***Pertinax***) ***quitensis*** (Kaup 1871) (Figs. 7, 8 and 9)

Type material. Holotype: sex. ind. **Ecuador**. Quito. *Proculejus quitensis* Kp. / Br. Mus. // Type // Quito // 46 62 (BMHN).

**Fig. 7:**
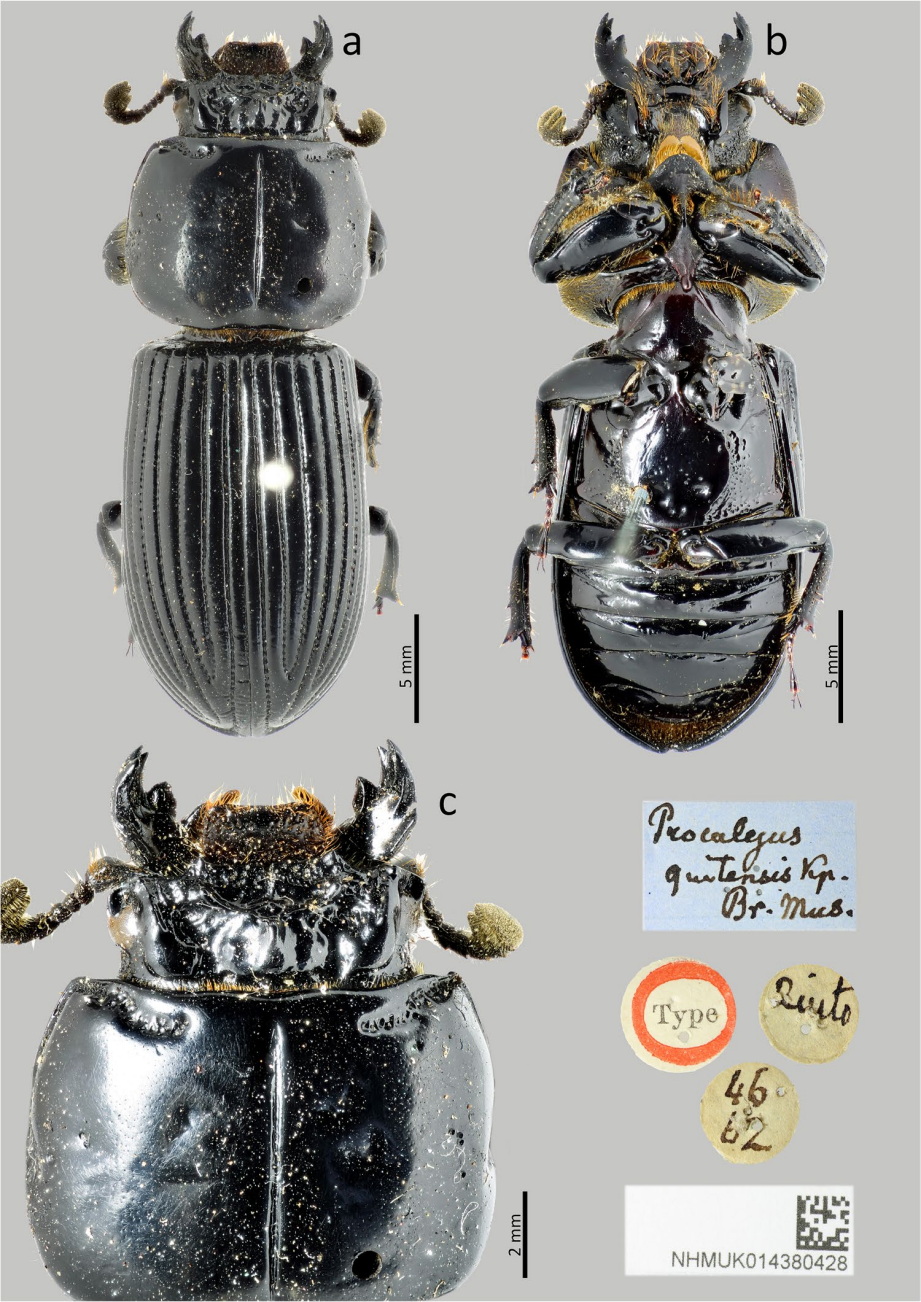
*Passalus* (*Pertinax*) *quitensis* type: (**a**) dorsal habitus; (**b**) ventral habitus; (**c**) dorsal view of the head and pronotum

**Fig. 8:**
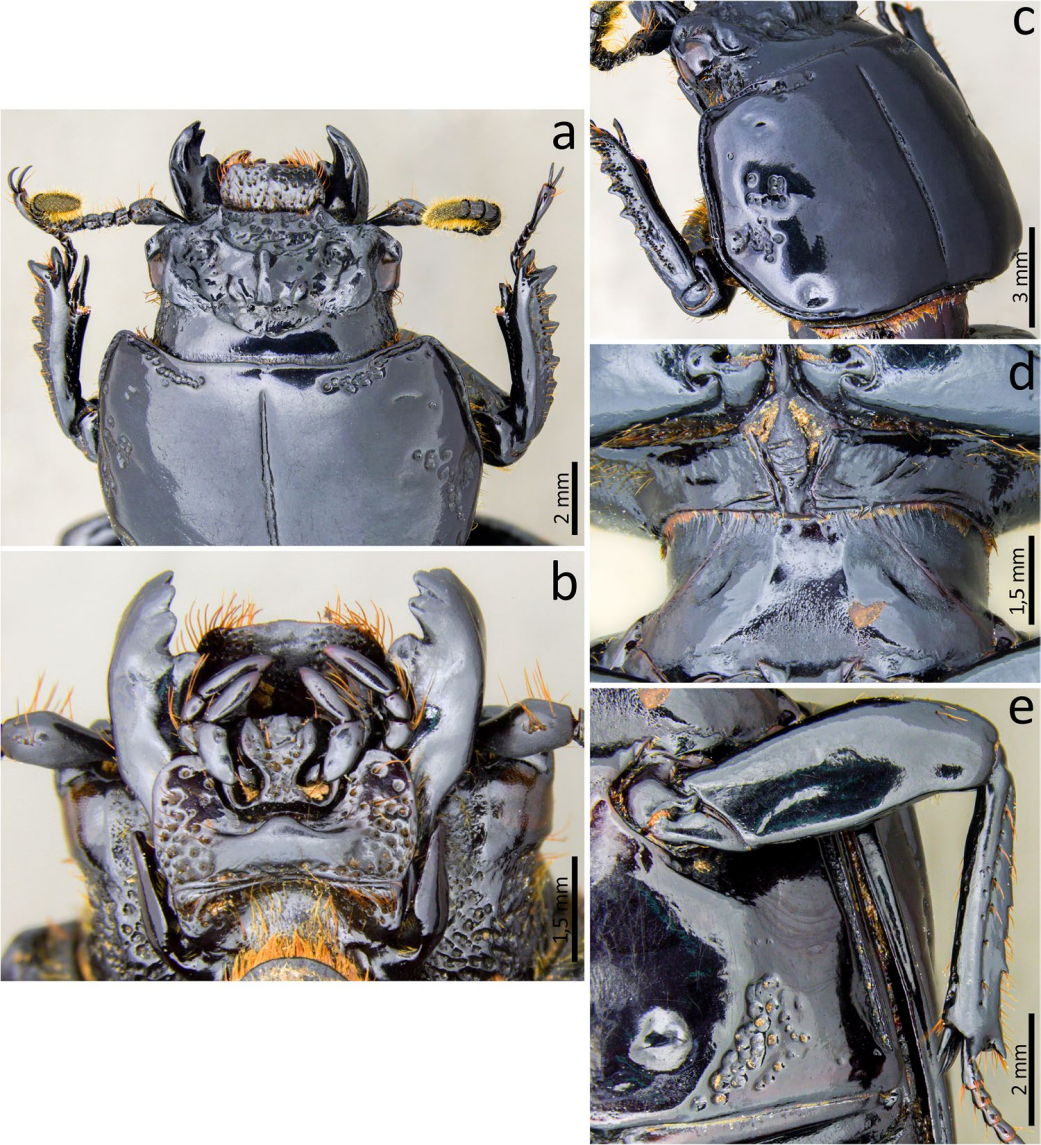
*Passalus* (*Pertinax*) *quitensis:* (**a**) head; (**b**) mentum; (**c**) head and pronotum in dorsolateral view; (**d**) mesosternum; (**e**) metasternum

**Fig. 9:**
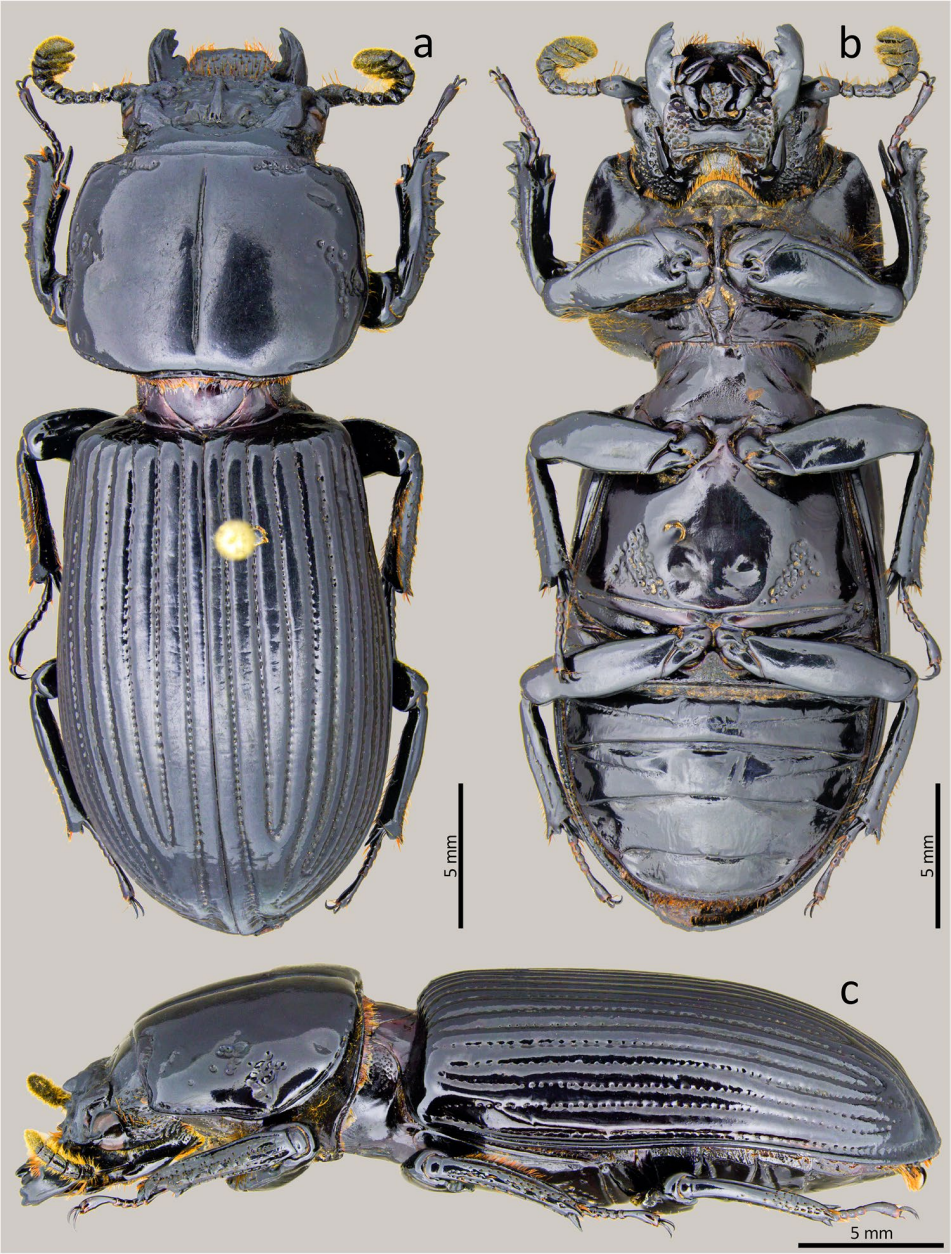
*Passalus* (*Pertinax*) *quitensis*: (**a**) dorsal habitus; (**b**) ventral habitus; (**c**) lateral habitus

Additional material: **Ecuador**. *Tung*[*urahua*], El Tablón (Baños) 2800 m. vii-2001. D. Curoe col. // *Passalus quitensis* (Kaup, 1871) / M. Bevilaqua det. 2019 (CEMT) 1 ex.

Diagnosis: Frons anterior edge straight, with middle indentation continuing in a longitudinal groove, without secondary mediofrontal tubercles; mediofrontal + laterofrontal tubercles large, inner tubercles large, smaller than the mediofrontal + laterofrontal tubercles; prepisternum with scarce pubescence; mesosternal scars large, narrow and shallow, with an opaque surface; metasternal disc short delimited by punctuations lateroposteriorly; humeri and epipleura glabrous.

Redescription.

Habitus (Figs. 7a-b and 9): total length 30 mm, brachypterous, body convex.

Head (Figs. 7c and 8a-b): labrum with anterior border straight. Clypeus hidden below the frons, anterior angles small, positioned below the mediofrontal + laterofrontal tubercles. Frons anterior edge straight, with middle indentation continuing in a longitudinal groove, without secondary mediofrontal tubercles. Mediofrontal + laterofrontal tubercles large, conspicuous, with acute apex and projected forward. Frontal area transverse, wider than long, sloping, full of thick punctuations. Inner tubercles large, conspicuous, with slightly rounded vertices, smaller than the mediofrontal + laterofrontal tubercles, from which they are separated, but located closer to them than to the central tubercle. Anterior frontal ridges absent. Posterior frontal ridges strong, high, starting at obtuse angle at the base of the central tubercle, having a small tubercle located close to the central tubercle. Cephalic mamelon small, conspicuous and not divided by a longitudinal groove. Central tubercle conical, tall, with apex free. Lateroposterior tubercles small, conspicuous, slightly rounded shape, located near the central tubercle but not joined to it by a ridge. Laterofrontal areas prominent, containing some thick punctuations. Lateropostfrontal areas with few punctures. Postfrontal groove well-marked, with notch in the middle region. Epicranial sutures well-marked. Epicranial fossae deep. Anterior head angles well-developed, with obtuse vertices, smaller than mediofrontal + laterofrontal tubercles. Ocular canthus narrow, straight, reaching halfway to the eye (eyes reduced). Postorbital fossae large, well-marked, deep, with punctures and some setae. Hypostomal process broad, glabrous and slightly close from mentum. Medial basal mentum slightly dilated, with few setigerous punctuations only in the posterior region; with protruded anterior region, without notch. Lateral lobes of the mentum outer face rounded, inner face straight, with sparse setae. Lateral fossae of mentum rounded, large, shallow, with few punctuations and setae. Antennal club trilamellate, with short, thick, and straight lamellae. Mandibles with incisor lobe with three well-formed teeth at the apex; robust upper internal tooth with a larger superior tubercle than the lower one; large and tall dorsal teeth, directed forward; inconspicuous infrabasal fossae. Maxilla with lacinia bidentate at the apex. Ligula tridentate, middle tooth slightly larger and the same width as the lateral teeth.

Thorax (Figs. 7, 8c-e and 9): pronotum rounded, punctures extended beyond the lateral fossae and marginal groove, same wide than elytra. Anterior edge slightly straight. Anterior angles obtuse. Marginal groove narrow, occupying 2/3 of the anterior margin of the pronotum, containing fine punctuations in the anterior and lateral part. Lateral fossae large, well-marked, deep, irregular in shape. Prepisternum with short setae located mainly in the lateroposterior region, the anterior region being glabrous. Prepimerum pubescence, mainly in the posterior region. Prosternellum rhomboidal, with rounded base. Mesosternum smooth and glabrous, with lateral areas opaque. Mesosternal scars large, narrow and shallow, with an opaque surface, without punctuations or setae. Posterior corner of the mesepisternum and mesepimere glabrous. Anterolateral part of metasternum and lateral fossa glabrous. Anterolateral part of metasternum and lateral fossa glabrous. Metasternal disc short and flat, with few punctures, delimited by a carina formed by punctuations in the lateroposterior region. Metasternal punctuations consisting in a group of thick punctures in the posterior region. Metasternal lateral fossea narrow, not dilated posteriorly, narrower than the mesotibia.

Elytra (Figs. 7a and 9a,c): striae narrower than interstriae; marked with rounded, shallow and inconspicuous punctuations on the dorsal striae, slightly more defined and deep on the lateral striae. Humeri and epipleura glabrous.

Abdomen (Figs. 7b and 9b): last sternite with marginal groove complete and well-marked.

Legs (Figs. 7a-b, 8a,e and 9): profemur with ventral anterior marginal sulcus well-marked and complete; posterior ventral edge with tuft of setae close to the apex. Protibiae not dilated. Mesotibiae with two or three small spines on the outer margin. Metatibiae without spines on the outer surface.

Comments: in the original description, Kaup (1871) made no reference to the species having reduced wings. However, Gravely (1918) indicates that the species possesses “rounded pronotum and fused and rounded elytra”, which corresponds to brachypterous species. Kaup (1871) only indicated as coming from Quito, which is located at more than 2500 m a.s.l.

### ***Passalus*** (***Pertinax***) ***striatissimus*** Luederwaldt, 1934 (Fig. 10)

Type material. Cotipo: sex. male. **Brazil**. [*Rio de Janeiro*] Itatiaya—160 m / Macieiras / 19.vii.1933 / J.F. Zikán // *Passalus striatissimus* Lüederw. / Lüederwaldt. 33 // 06,510 (MZUSP).

**Fig. 10:**
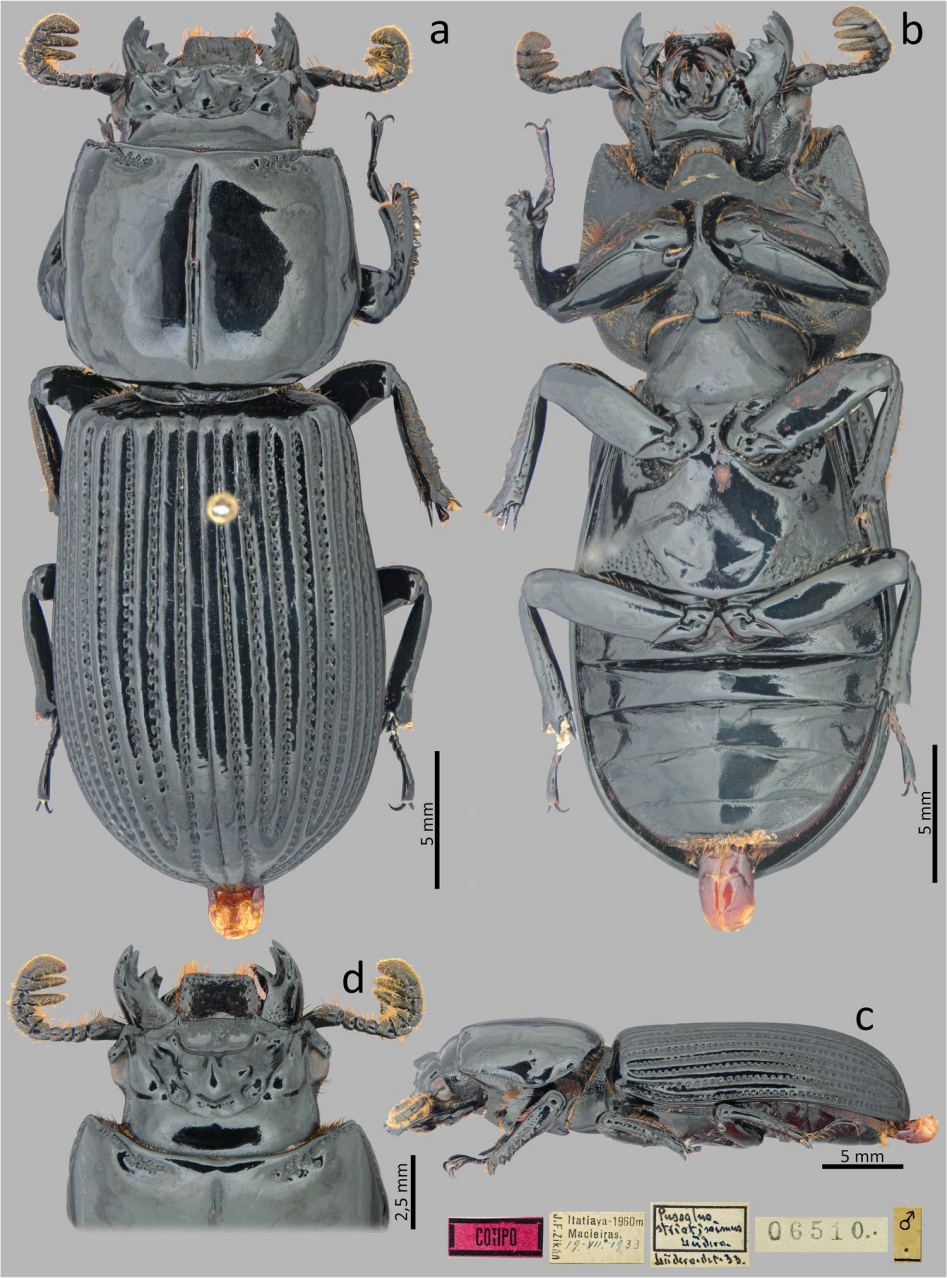
*Passalus* (*Pertinax*) *striatissimus*: (**a**) dorsal habitus; (**b**) ventral habitus; (**c**) lateral habitus; (**d**) head and anterior part of pronotum

Diagnosis: Frons anterior edge straight or slightly convex, without middle indentation; frontal area fully punctuated on anterior region; mediofrontal + laterofrontal tubercles smaller than inner tubercles; posterior frontal ridges strong, high, starting transversely at the base of the central tubercle; mesosternal scars large, narrow, and shallow, with an opaque surface; metasternum posteriorly bounded by a large group of thick punctures over the lateroposterior region; epipleura glabrous; humeri with short tuft of setae.

Redescription.

Habitus (Figs. 10a-c): total length 30 to 31 mm, brachypterous, body convex.

Head (Figs. 10b,d): labrum with anterior border straight. Clypeus hidden below the frons, anterior angles small and acute, positioned below the mediofrontal + laterofrontal tubercles. Frons anterior edge straight or slightly convex, without middle indentation, without secondary mediofrontal tubercles. Mediofrontal + laterofrontal tubercles small, conspicuous, projected forward with acute apex. Frontal area transverse, twice wider than long, sloping, with the tegument of the anterior region full of thick punctuations and smooth posterior area. Inner tubercles large, conspicuous with slightly obtuse vertices, larger than mediofrontal + laterofrontal tubercles, from which they are separated, but located closer to them than to the central tubercle. Anterior frontal ridges absent. Posterior frontal ridges strong, high, starting transversely at the base of the central tubercle. Cephalic mamelon small, inconspicuous, slightly triangular in shape and not divided by a longitudinal groove. Central tubercle conical, not flat, tall, with apex no free. Lateroposterior tubercles small, conspicuous, located far from the central tubercle, without a keel connecting them. Lateropostfrontal area deep, with smooth surface. Postfrontal groove well-marked, with notch in the middle region. Anterior head angles well-developed, with acute vertices, larger than mediofrontal + laterofrontal tubercles. Canthus ocular narrow, apex straight or slightly rounded, almost reaching the middle of the eye (eyes reduced). Hypostomal process wide, glabrous, and distant from the mentum. Medial basal mentum slightly dilated, without setigerous punctuations; with protruded anterior region and middle notched. Lateral fossae of the mentum oval, open, large, deep, without punctures or setae. Antennal club trilamellate, with short, robust, and straight lamellae. Mandibles with incisor lobe with three well-formed teeth at the apex; robust upper internal tooth with a larger superior tubercle than the lower one; large and tall dorsal teeth, directed forward; inconspicuous infrabasal fossae. Maxilla with lacinia bidentate at the apex. Ligula tridentate with medium tooth slightly larger and with the same width as the lateral teeth.

Thorax (Fig 10): pronotum same wide of elytra, anterior border slight concave; anterior angles straight. Marginal groove narrow, occupying ½ of the anterior margin of the pronotum, containing fine punctuations in the anterior and lateral region. Lateral fossae large, well-marked, deep, irregular in shape, with scarce punctures (less than 5). Prepimerum with sparse pubescence, mainly in the posterior region. Prosternelum rhomboidal, with a constriction close to the base, which is truncated. Mesosternum smooth and glabrous, with the tegument of the lateral areas opaque. Mesosternal scars large, narrow, and shallow, with an opaque surface, without punctures or setae. Posterior corner of the mesepisternum and mesepimere glabrous. Anterolateral part of metasternum and lateral fossa glabrous. Metasternal disc without punctures, posteriorly bounded by a carina formed by a large group of thick punctures over the lateroposterior region. Metasternal lateral fossae glabrous, narrow, not dilated at the apex; narrower than mesotibia.

Elytra (Figs. 10a, b, c): epipleura glabrous. Humeri with short tuft of setae. Striae narrower than interstriae; marked with rounded, deep and conspicuous punctures on the dorsal and lateral striae.

Abdomen (Fig. 10b): Last sternite with marginal groove incomplete and well-marked.

Legs (Figs. 10a, b, c): profemur with ventral anterior marginal sulcus marked and complete. Protibiae not dilated. Mesotibiae with two or three small spines on the outer margin. Metatibiae without spines on the outer surface.

Aedeagus elongated (Figs. 10a, b). Basal piece distinctly separated from the parameres. Median lobe practically the same width as the basal piece and the parameres; in ventral view with two large plaques, very sclerotized. Parameres in ventral view with a recess in the median region in a “V” shape; transverse anterior margin; in side view with apexes of the narrow projections not reaching half the length of median lobe; in dorsal view projections not joined.

### ***Passalus*** (***Pertinax***) ***sulcifrons*** (Kuwert, 1898) (Figs. 11 and 12)

Material examined: sex. female. **Ecuador**. Pichincha—Reserva Geobotánica Pululahua. ii.1999 / Col. María de los Angeles Simbaña R. Colecta manual en los senderos de la Reserva // *Pertinax sulcifrons* Kuw. 1898 / S. Boucher det. [20]14 (CEMT) 1 ex.

**Fig. 11:**
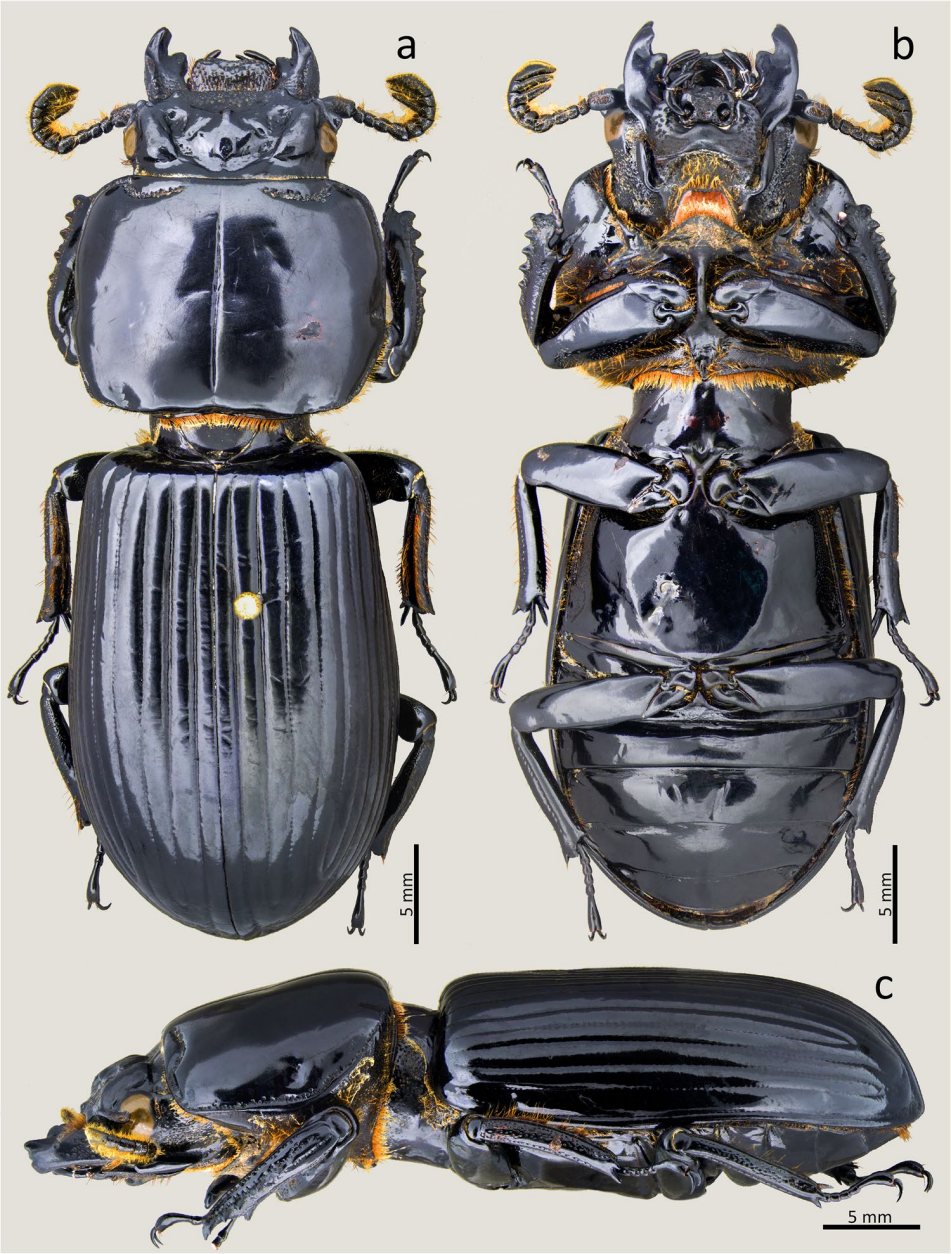
*Passalus* (*Pertinax*) *sulcifrons*: (**a**) dorsal habitus; (**b**) ventral habitus; (**c**) lateral habitus

**Fig. 12:**
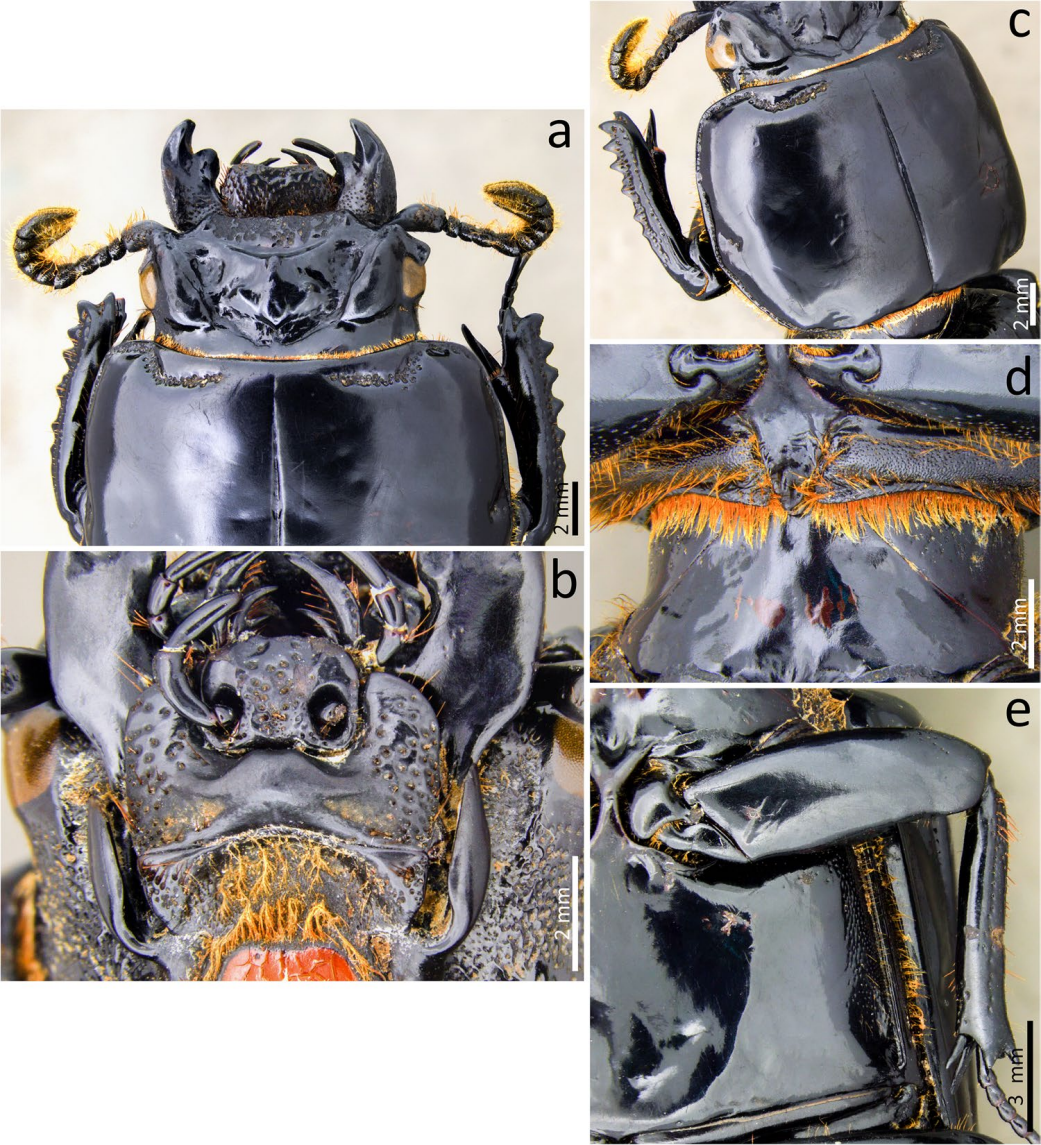
*Passalus* (*Pertinax*) *sulcifrons:* (**a**) head; (**b**) mentum; (**c**) head and pronotum in dorsolateral view; (**d**) mesosternum; (**e**) metasternum

Diagnosis: Frons wide, entirely covered by thick punctures; mediofrontal + laterofrontal tubercles small, but larger than inner tubercles, from which they are separated; mesosternal scars absent or inconspicuous; posterior corner of the mesepisternum and mesepimere finely pubescent; setigerous punctuations in the anterior region and in the metasternal fossae, with pubescence composed of sparse and slightly short setae in the anterior region.

Redescription.

Habitus (Fig. 11): total length 43.0–49.5 mm, brachypterous, body convex.

Head (Figs. 12a,b): labrum with anterior border slightly concave. Clypeus hidden below the frons, anterior angles small, positioned below the mediofrontal + laterofrontal tubercles. Frons wide, entirely covered by thick punctures, without secondary mediofrontal tubercles. Mediofrontal + laterofrontal tubercles small, conspicuous, with obtuse apexes and projected forward. Inner tubercles small, smaller than the mediofrontal + laterofrontal tubercles, from which they are separated. Anterior frontal ridges weak, just close to internal tubercles, disappearing before reaching mediofrontal + laterofrontal tubercles. Posterior frontal ridges strong, low, starting at obtuse angle at the base of the central tubercle. Cephalic mamelon absent. Central tubercle conical, flattened, low posteriorly, with only the apex evident, but not free. Lateroposterior tubercles small, conspicuous, with acute and very evident apex; distant from the central tubercle and not joined to it by a keel. Laterofrontal areas shallow with a smooth surface or containing few fine punctures. Lateropostfrontal areas deep, glabrous, and sparsely punctuated. Anterior head angles well-developed, with obtuse vertices, smaller than mediofrontal + laterofrontal tubercles. Canthus ocular wide, with straight anterior angles, not reaching halfway to the eye. Hypostomal process broad, glabrous and slightly distant from mentum. Medial basal mentum slightly dilated, not punctuated, with protruded anterior region and notched. Mentum with big lateral fossae, oval, shallow, and some setae on the outer surface. Antennal club trilamellate, with short, thick, and straight lamellae. Mandibles with incisor lobe with three well-formed teeth at the apex. Maxilla with lacinia bidentate at the apex. Ligula tridentate with medium tooth slightly larger and with the same width as the other two lateral teeth.

Thorax (Figs. 11 and 12c-e): pronotum rounded, same wide than elytra. Anterior edge slightly concave. Anterior angles obtuse. Marginal groove narrow, occupying ¾ of the anterior margin of the pronotum, containing thick punctures. Lateral fossae large, well-marked, deep, with about 3 punctures. Prosternum with thin and slightly dense pubescence in the lateroposterior region, consisting of long setae and dense pubescence (short setae), mainly in the posterior region, practically not visible in the dorsal view. Prosternellum rhomboidal, with acute base. Mesosternum smooth and glabrous, with lateral areas tegument opaque. Mesosternal scars absent or inconspicuous. Posterior corner of the mesepisternum and mesepimere finely pubescent. Anterolateral part of metasternum and lateral fossa glabrous or finely pubescent. Setigerous punctuations in the anterior region and in the metasternal fossae, with pubescence composed of sparse and slightly short setae in the anterior region close to the mesocoxae and in the metasternal fossae. Metasternal disc short and flat, no punctuated. Metasternal punctuations absent. Metasternal lateral fossae large, dilated at the apex, almost the same width as the mesotibiae.

Elytra (Fig. 11): humeri and epipleura glabrous. Striae narrower than interstriae, without punctuation on the dorsal striae and with shallow and inconspicuous punctuation on the lateral striae.

Abdomen (Fig. 11c): last sternite with marginal groove incomplete and well-marked.

Legs (Fig. 11): profemur with ventral anterior marginal sulcus weakly marked and complete; posterior ventral edge with tuft of setae close to the apex. Protibiae not dilated. Mesotibiae and metatibiae without spines on the outer surface.

Aedeagus elongated. Basal piece slightly narrower and longer than the parameres, distinctly separated from the parameres. Median lobe narrower than the parameres; in ventral view with two sclerotized plaques covering almost the entire ventral region. Parameres in ventral view, medially separated; concave anterior margin; in lateral view with slightly rounded projections, not reaching half the length of the median lobe; in dorsal view unjoined projections.

Comments**:** Boucher (1990) revalidates this species, indicating it is brachypterous. Likewise, this author indicates the species is endemic to the Andes, from around 2000 m a.s.l.

### ***Passalus*** (***Pertinax***) ***bolivianus*** Jiménez-Ferbans, Reyes-Castillo & Schuster, 2019

Diagnosis: Mediofrontal tubercles larger than inner tubercles; lateroposterior tubercles larger than central tubercle; anterior border of frons almost straight with small middle indentation; elytral humeri heavily pubescent.

Known from Bolivia, described as brachypterous based on material from 1800–3200 m a.s.l.

### ***Passalus*** (***Pertinax***) ***canoi*** Jiménez-Ferbans, Reyes-Castillo & Schuster, 2019 (Fig. 13)

Diagnosis: Strong indentation on frontal edge, internal tubercles joined to mediofrontal tubercles by a weak ridge, antennal club with robust, long, and curved inwards lamellae, humeri and epipleura glabrous, inferolateral area of pronotum with sparse pubescence, and metasternal disc delimited by punctures only posteriorly.

**Fig. 13:**
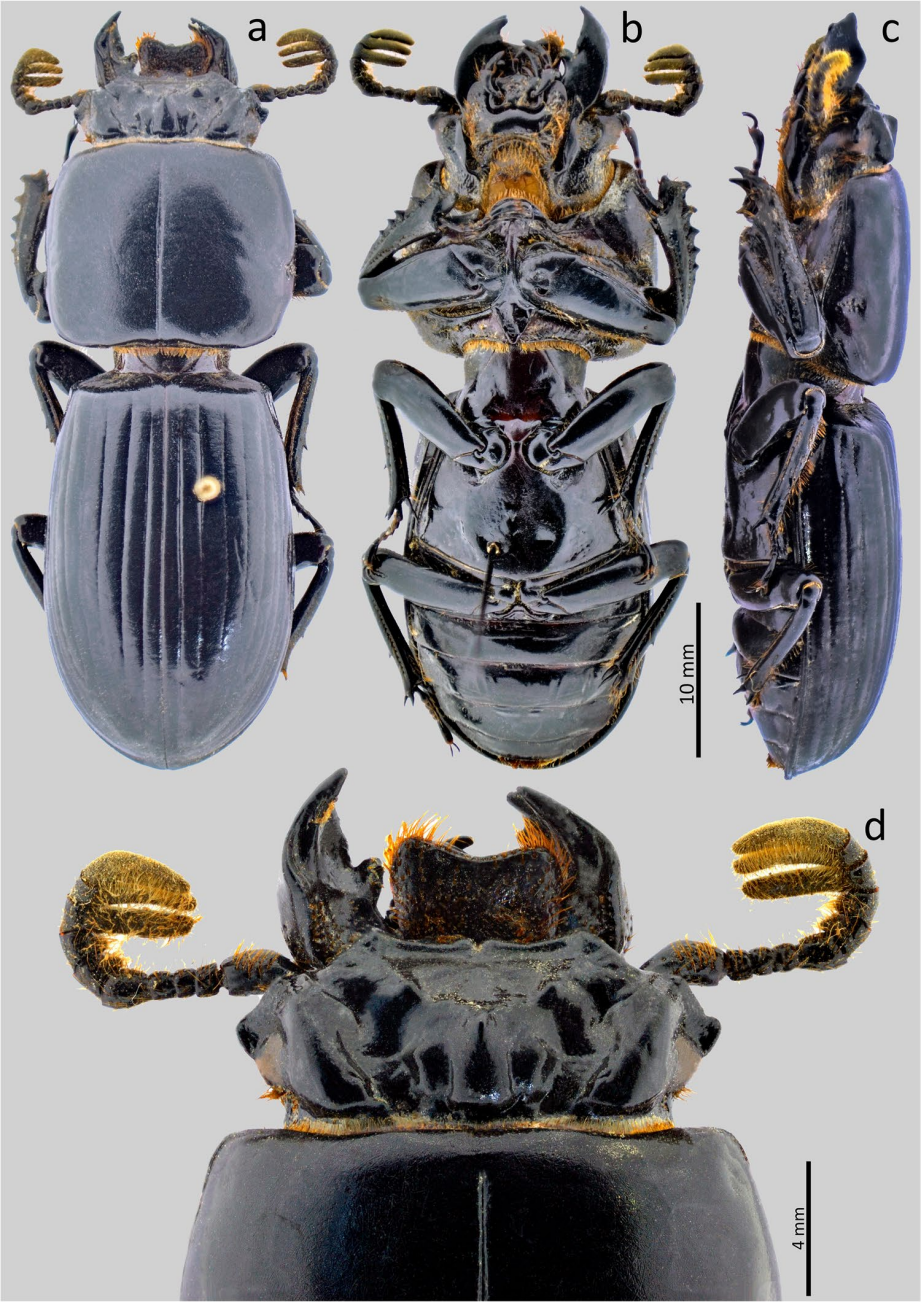
*Passalus* (*Pertinax*) *canoi*: (**a**) dorsal habitus; (**b**) ventral habitus; (**c**) lateral habitus; (**d**) dorsal view of the head and anterior part of pronotum

Described as brachypterous from Bolivia, based on two female specimens from 2000 m a.s.l. Bevilaqua and Fonseca (in prep.) complements the description with the male genitalia.

### ***Passalus*** (***Pertinax***) ***connatus*** Luederwaldt, 1941

Diagnosis: Anterior frontal edge straight without secondary mediofrontal tubercles; mediofrontal area slightly transverse and punctuated anteriorly; robust central tubercle with slightly free apex; mediofrontal + laterofrontal tubercles similar and close to inner tubercles; strong posterior frontal ridges; narrow ocular canthus with rounded apex; anterior pronotum angles rounded; anterior marginal groove at 2/3 of the pronotal width; prepisternum with conspicuous setae; pubescent humeri and epipleura; metasternum densely punctuated lateroposteriorly; pubescent metasternal lateral groove.

Remarks: Indicated by the author as fused elytra, in the description only one specimen from Mexico is cited, without further details. We examined the holotype, only labeled “Mexico” and deposited in the MZUSP-Brazil collection. It presents as striking characteristics pubescent humeri and reduced wings, common characteristics in some Andean *Passalus* of Colombia and Ecuador. In the original description, Luederwaldt (1941) mentioned its proximity to *Passalus affinis* Percheron 1835, an endemic species to Hispaniola (Reyes-Castillo et al. 1995; Jiménez-Ferbans et al. 2015), from which it is distinguished by presenting the “élitros soldados” (soldered elytra). Reyes-Castillo (1985) cited *P. connatus* from Mexico, however, we doubt that it is found in that country, where to date a brachypterous *Passalus* has never been collected. We did not examinate the wing, but its habitus suggests it is hemibrachypterous.

### ***Passalus*** (***Pertinax***) ***gonzalezae*** Jiménez-Ferbans, Reyes-Castillo & Schuster, 2019

Diagnosis: Anterior frontal edge straight, with a notch in the median region; latero + mediofrontal tubercles larger than inner tubercles; mediofrontal area, as long as wide, with three grooves forming an inverted “Y”; prepisternum with sparse pubescence; mesosternal scars elongated, wide, shallow and with opaque surface; metasternum punctuated posteriorly; metasternal lateral groove narrow and glabrous; epipleura and humeri glabrous; weak punctures on elytral striae 7–10.

This species was described from Bolivia, based on one female from 2000 m a.s.l. Bevilaqua and Fonseca (in prep.) complements the description with the male genitalia. It is brachypterous (Fig. 3c).

### ***Passalus*** (***Pertinax***) ***halffterorum*** Jiménez-Ferbans, Reyes-Castillo, Schuster & Beza-Beza, 2017

Diagnosis: Anterior frontal edge with small middle indentation, without secondary mediofrontal tubercles; mediofrontal + laterofrontal tubercles, smaller and joined to inner tubercles, by a weak ridge; canthus ocular wide, eyes reduced; pronotum with acute anterior; mesosternum without mesosternal scar, indicated only by an opaque anterior area; humeri and epipleura with scarce minute setae.

Described from Costa Rica, this species is known from elevations between 2600 and 3075 m.

### ***Passalus*** (***Pertinax***) ***nodifrons*** Dibb, 1948

Diagnosis: Anterior frontal edge of head slightly concave, lacking notch medialy; mediofrontal area with rough surface; cephalic nodule large; inner tubercles large, but smaller than the latero + mediofrontal tubercles, from which are separate; anterofrontal ridges absent, posterofrontal ridges straight and strong; prepisternum with scarce pubescence; mesosternal scars absent; epipleura and humeri glabrous.

Remarks: Dibb (1948) described this species based on specimens from “Bolivia: La Paz”, indicating it has “elytra soldered”. Until now, nobody has cited more specimens of this species. In MZUSP there is a specimen which meets the original description, with the following label: Peru. Boqueron. Loreto. VIII.[1]948 / *Passalus nodifrons* Dibb, P. Pereira det. 1967. In 1948 the Department of Loreto was larger and later it was divided into Loreto and Ucayali. In Ucayali there is a place named Boquerón del Padre Abad, it is a canyon in a mountain that reaches 1300 m a.s.l. Bevilaqua and Fonseca (in prep.) redescribe the species based on the original type material and it is hemibrachypterous.

### ***Passalus*** (***Pertinax***) ***rzedowskiorum*** Jiménez-Ferbans, Reyes-Castillo, Schuster & Beza-Beza, 2017

Diagnosis: Anterior frontal edge notched, without secondary mediofrontal tubercles; mediofrontal + laterofrontal tubercles larger and joined to inner tubercles by a weak ridge, lateropostfrontal with conspicuous striae; pronotum with anterior angles acute; mesosternal scar slightly marked, oval shaped; metasternum medially and posteriorly punctuated; humeri and epipleura glabrous.

This species was described as hemibrachypterous. So far it is known only for the type material from 1480 to 2700 m a.s.l.

## Discussion

Although Percheron (1835) and Kaup (1871) commented on the shape of the body and elytra in the brachypterous species, Bates (1886) was the first to use this character to classify species in Central America. The genera were grouped into two sections, characterized by the shape of the base of the elytra and its length relative to the thorax. This classification was criticized by subsequent authors (Gravely 1918; Hincks 1933) since these characters do not reflect the relationships between the different lineages, but instead are more related to the habits of the species. Thus, as far as is known, in the New World all the brachypterous species inhabit mountains. However, for some groups of the genus level, this condition is shared by all species and could represent a homologous state (v.g., *Ogyges*, *Proculejus*, and *Proculus*, *Veturius* (*Publius*)).

**Fig. 14:**
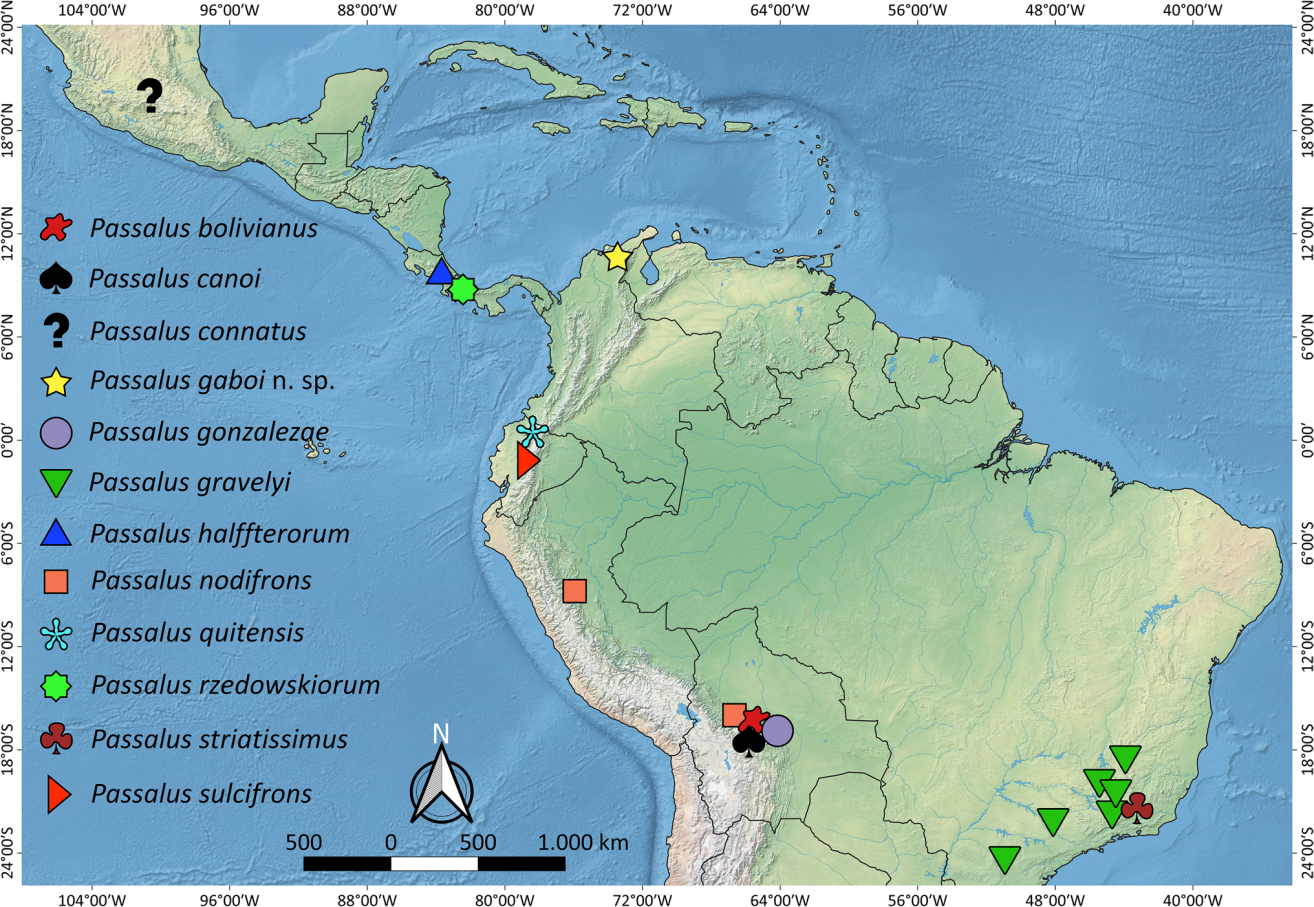
Distribution of brachypterous species of *Passalus* (*Pertinax*)

In *Passalus*, the brachypterous species do not exhibit a sister group relationship, and the condition in the genus may be more related to adaptations to life in the mountains. In the *P*. (*Passalus*) group ‘Petrejus’, for example, a high percentage of the species that live above 2000 m a.s.l. show some degree of wing reduction. In the case of *Passalus* (*Pertinax*), except for *P. connatus* (in which the original description only cited one specimen from Mexico, without further details of locality), all the brachypterous species inhabit high mountain areas in Central and South America (Fig. 14). However, Boucher (1990) comments that the habitus, median frontal architecture, and brachypterism, as well as the geographic distribution and altitude suggest a natural group of the “Andean brachypterous *Pertinax*”, different of the “macropterous *P*. *convexus* group” making this group morphologically, ecologically, and biogeographically specific to the entire subgenus.

The knowledge about the condition and microscopic morphology of the wing and related structures is scarce. In addition to the inability to fly and other external features, passalids with brachypterism generally have short elytra, enlarged pronotum and a general rounded body-shape (Reyes-Castillo 1970; Boucher 2006). These morphofunctional characters are very important in the evolution of lineages and, therefore, in their systematics. However, according to Boucher (2006), the characters related to wing reduction are not currently used due to their polymorphic nature.

Recently, Ariza-Marín and Amat-García (2021) tested the relationship between elevation and morphological changes in hind wings and elytra. According to them, for some genera of Proculini in Colombia, changes in elevation are directly related to differences in the shape of hind wings and elytra, suggesting that elevation produces a simultaneous change in both wings in Passalidae, making them smaller and more rounded. In addition to wing morphology, brachypterism in Passalidae is also accompanied with other morphological modifications as metasternal fossae reduction, reduction in overall body punctuation (although this is not a character that is present in all or most species of reduced wings), and eye reduction (Reyes-Castillo 1970). However, so far very few authors have documented systematically the brachypterism in Passalidae, for example (Reyes-Castillo 1970; Boucher 2006; Hincks 1933), and the causes and mechanisms that regulate this condition are unknown.
